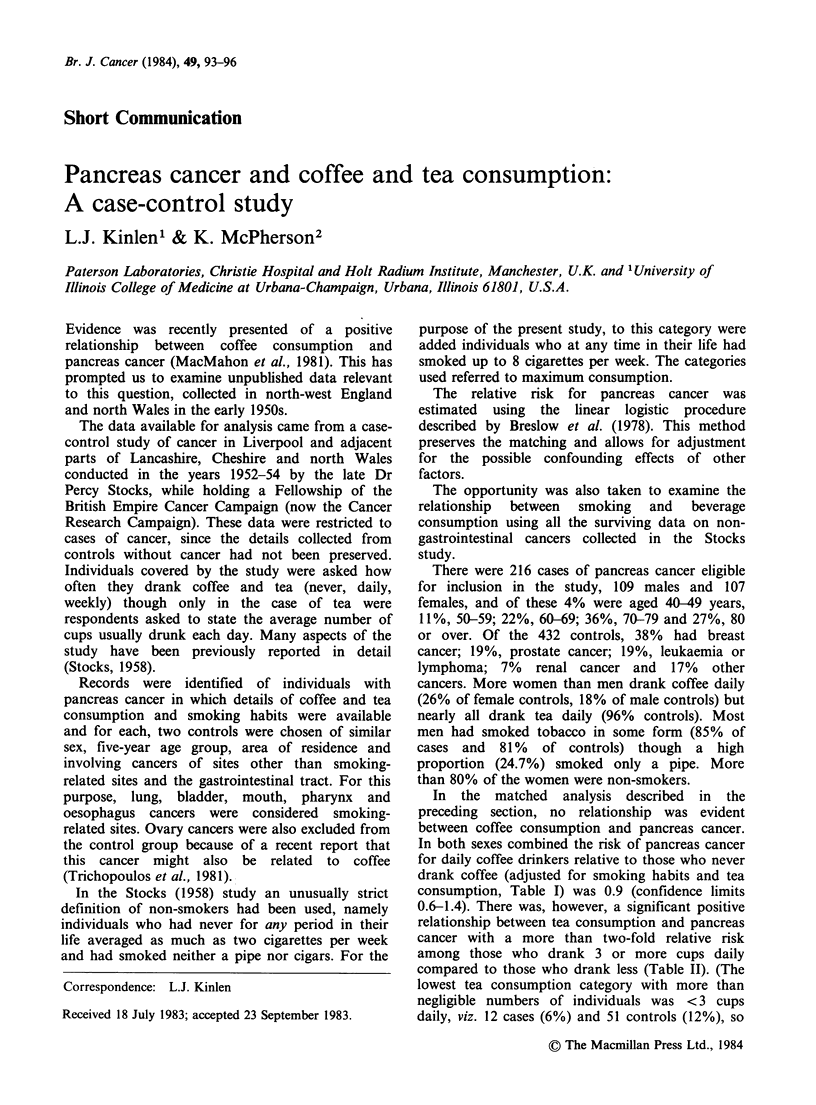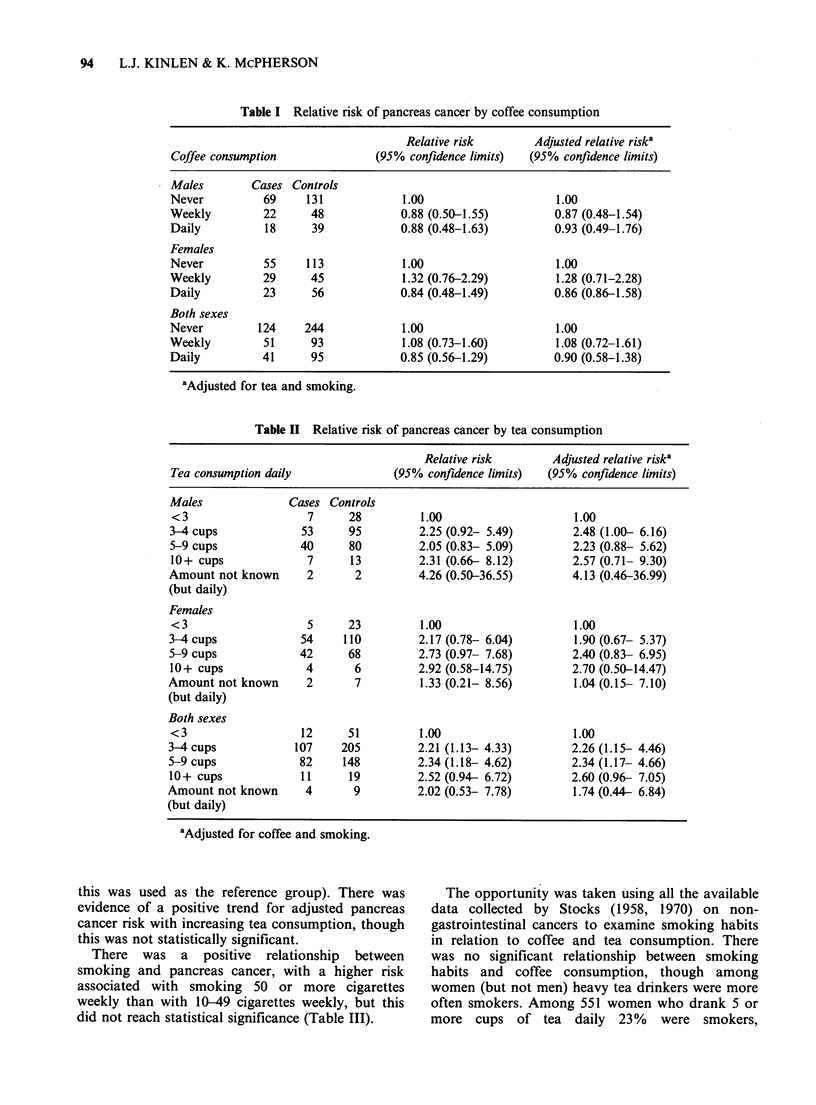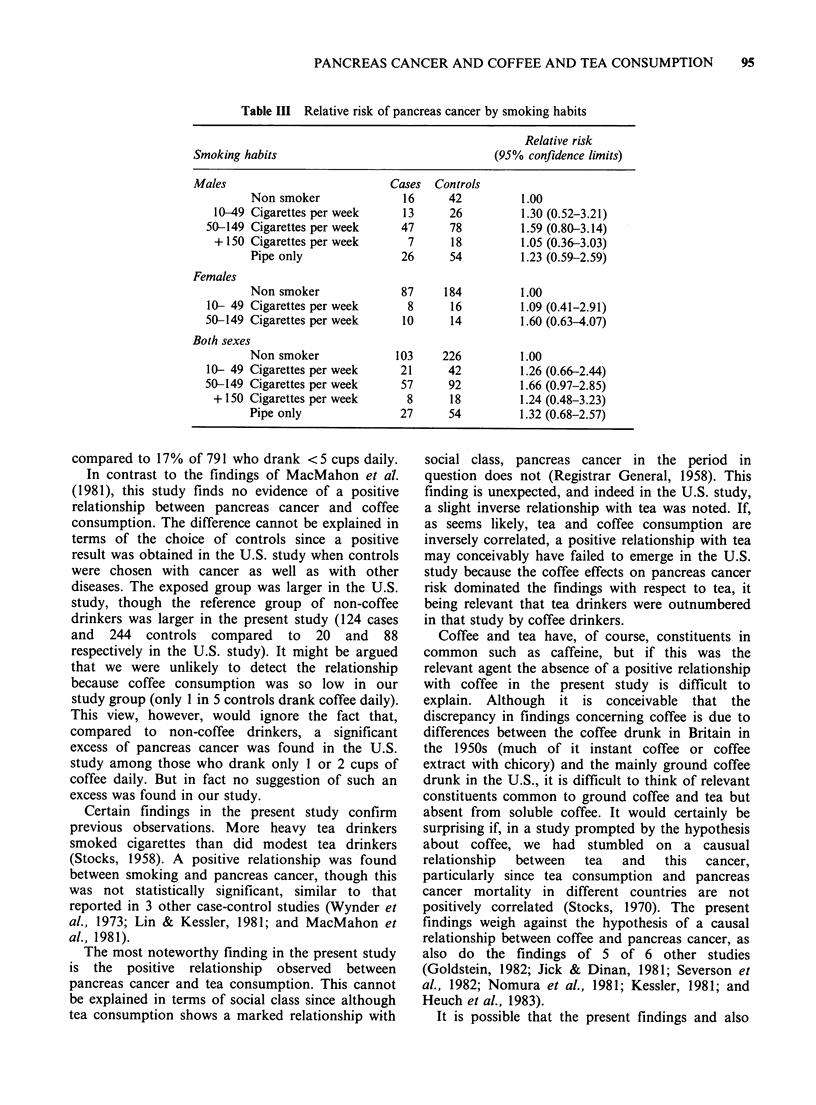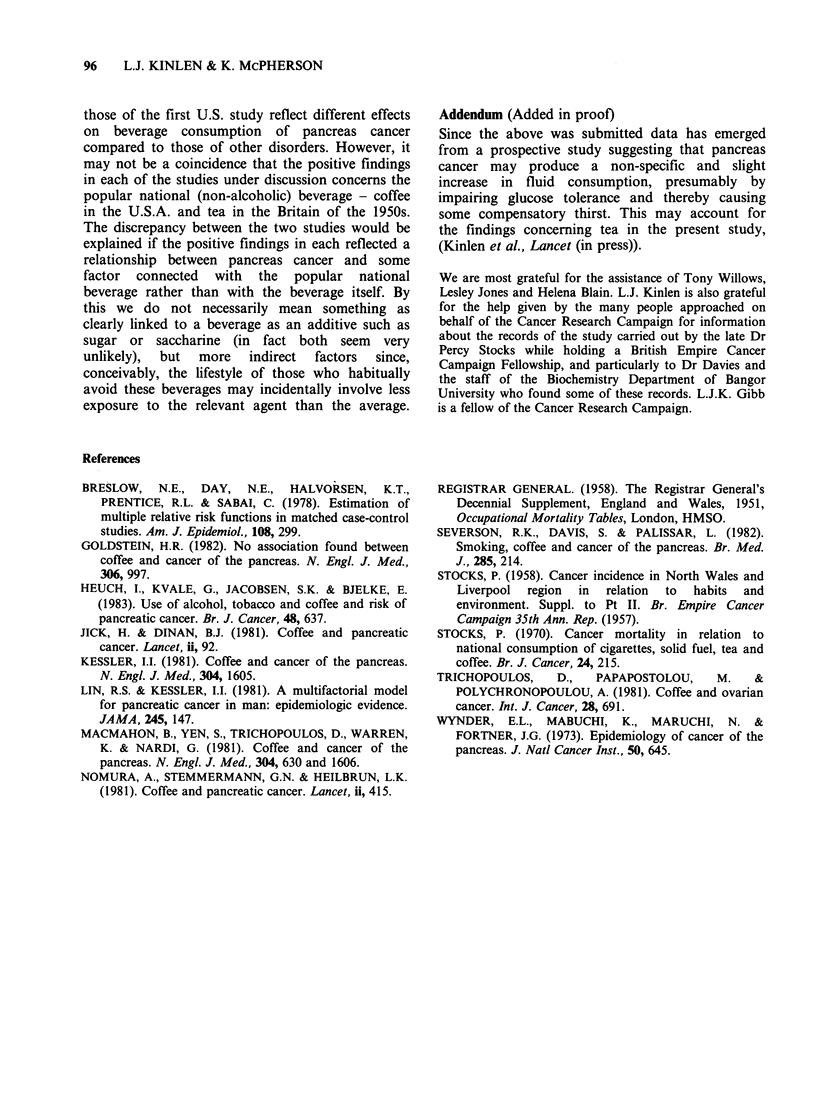# Pancreas cancer and coffee and tea consumption: a case-control study.

**DOI:** 10.1038/bjc.1984.14

**Published:** 1984-01

**Authors:** L. J. Kinlen, K. McPherson


*Br. J. Cancer* (1984), **49**, 93–96

### Short Communication

### Pancreas cancer and coffee and tea consumption: A case-control study

L.J. Kinlen[1] & K. McPherson[2]

*Paterson Laboratories, Christie Hospital and Holt Radium Institute, Manchester, U.K. and [1]University of Illinois College of Medicine at Urbana-Champaign, Urbana, Illinois 61801, U.S.A.*

Evidence was recently presented of a positive relationship between coffee consumption and pancreas cancer (MacMahon *et al.*, 1981). This has prompted us to examine unpublished data relevant to this question, collected in north-west England and north Wales in the early 1950s.

The data available for analysis came from a case-control study of cancer in Liverpool and adjacent parts of Lancashire, Cheshire and north Wales conducted in the years 1952–54 by the late Dr Percy Stocks, while holding a Fellowship of the British Empire Cancer Campaign (now the Cancer Research Campaign). These data were restricted to cases of cancer, since the details collected from controls without cancer had not been preserved. Individuals covered by the study were asked how often they drank coffee and tea (never, daily, weekly) though only in the case of tea were respondents asked to state the average number of cups usually drunk each day. Many aspects of the study have been previously reported in detail (Stocks, 1958).

Records were identified of individuals with pancreas cancer in which details of coffee and tea consumption and smoking habits were available and for each, two controls were chosen of similar sex, five-year age group, area of residence and involving cancers of sites other than smoking-related sites and the gastrointestinal tract. For this purpose, lung, bladder, mouth, pharynx and oesophagus cancers were considered smoking-related sites. Ovary cancers were also excluded from the control group because of a recent report that this cancer might also be related to coffee (Trichopoulos *et al.*, 1981).

In the Stocks (1958) study an unusually strict definition of non-smokers had been used, namely individuals who had never for *any* period in their life averaged as much as two cigarettes per week and had smoked neither a pipe nor cigars. For the purpose of the present study, to this category were added individuals who at any time in their life had smoked up to 8 cigarettes per week. The categories used referred to maximum consumption.

The relative risk for pancreas cancer was estimated using the linear logistic procedure described by Breslow *et al.* (1978). This method preserves the matching and allows for adjustment for the possible confounding effects of other factors.

The opportunity was also taken to examine the relationship between smoking and beverage consumption using all the surviving data on non-gastrointestinal cancers collected in the Stocks study.

There were 216 cases of pancreas cancer eligible for inclusion in the study, 109 males and 107 females, and of these 4% were aged 40–49 years, 11%, 50–59; 22%, 60–69; 36%, 70–79 and 27%, 80 or over. Of the 432 controls, 38% had breast cancer; 19%, prostate cancer; 19%, leukaemia or lymphoma; 7% renal cancer and 17% other cancers. More women than men drank coffee daily (26% of female controls, 18% of male controls) but nearly all drank tea daily (96% controls). Most men had smoked tobacco in some form (85% of cases and 81% of controls) though a high proportion (24.7%) smoked only a pipe. More than 80% of the women were non-smokers.

In the matched analysis described in the preceding section, no relationship was evident between coffee consumption and pancreas cancer. In both sexes combined the risk of pancreas cancer for daily coffee drinkers relative to those who never drank coffee (adjusted for smoking habits and tea consumption, Table I) was 0.9 (confidence limits 0.6–1.4). There was, however, a significant positive relationship between tea consumption and pancreas cancer with a more than two-fold relative risk among those who drank 3 or more cups daily compared to those who drank less (Table II). (The lowest tea consumption category with more than negligible numbers of individuals was <3 cups daily, *viz.* 12 cases (6%) and 51 controls (12%), so

Correspondence: L.J. Kinlen

Received 18 July 1983; accepted 23 September 1983.

© The Macmillan Press Ltd., 1984

**Table I** Relative risk of pancreas cancer by coffee consumption

| *Coffee consumption* | | | *Relative risk (95% confidence limits)* | *Adjusted relative risk*[a] *(95% confidence limits)* |
|---|---|---|---|---|
| *Males* | *Cases* | *Controls* | | |
| Never | 69 | 131 | 1.00 | 1.00 |
| Weekly | 22 | 48 | 0.88 (0.50–1.55) | 0.87 (0.48–1.54) |
| Daily | 18 | 39 | 0.88 (0.48–1.63) | 0.93 (0.49–1.76) |
| *Females* | | | | |
| Never | 55 | 113 | 1.00 | 1.00 |
| Weekly | 29 | 45 | 1.32 (0.76–2.29) | 1.28 (0.71–2.28) |
| Daily | 23 | 56 | 0.84 (0.48–1.49) | 0.86 (0.86–1.58) |
| *Both sexes* | | | | |
| Never | 124 | 244 | 1.00 | 1.00 |
| Weekly | 51 | 93 | 1.08 (0.73–1.60) | 1.08 (0.72–1.61) |
| Daily | 41 | 95 | 0.85 (0.56–1.29) | 0.90 (0.58–1.38) |

[a]Adjusted for tea and smoking.

**Table II** Relative risk of pancreas cancer by tea consumption

| *Tea consumption daily* | | | *Relative risk (95% confidence limits)* | *Adjusted relative risk*[a] *(95% confidence limits)* |
|---|---|---|---|---|
| *Males* | *Cases* | *Controls* | | |
| <3 | 7 | 28 | 1.00 | 1.00 |
| 3–4 cups | 53 | 95 | 2.25 (0.92– 5.49) | 2.48 (1.00– 6.16) |
| 5–9 cups | 40 | 80 | 2.05 (0.83– 5.09) | 2.23 (0.88– 5.62) |
| 10+ cups | 7 | 13 | 2.31 (0.66– 8.12) | 2.57 (0.71– 9.30) |
| Amount not known (but daily) | 2 | 2 | 4.26 (0.50–36.55) | 4.13 (0.46–36.99) |
| *Females* | | | | |
| <3 | 5 | 23 | 1.00 | 1.00 |
| 3–4 cups | 54 | 110 | 2.17 (0.78– 6.04) | 1.90 (0.67– 5.37) |
| 5–9 cups | 42 | 68 | 2.73 (0.97– 7.68) | 2.40 (0.83– 6.95) |
| 10+ cups | 4 | 6 | 2.92 (0.58–14.75) | 2.70 (0.50–14.47) |
| Amount not known (but daily) | 2 | 7 | 1.33 (0.21– 8.56) | 1.04 (0.15– 7.10) |
| *Both sexes* | | | | |
| <3 | 12 | 51 | 1.00 | 1.00 |
| 3–4 cups | 107 | 205 | 2.21 (1.13– 4.33) | 2.26 (1.15– 4.46) |
| 5–9 cups | 82 | 148 | 2.34 (1.18– 4.62) | 2.34 (1.17– 4.66) |
| 10+ cups | 11 | 19 | 2.52 (0.94– 6.72) | 2.60 (0.96– 7.05) |
| Amount not known (but daily) | 4 | 9 | 2.02 (0.53– 7.78) | 1.74 (0.44– 6.84) |

[a]Adjusted for coffee and smoking.

this was used as the reference group). There was evidence of a positive trend for adjusted pancreas cancer risk with increasing tea consumption, though this was not statistically significant.

There was a positive relationship between smoking and pancreas cancer, with a higher risk associated with smoking 50 or more cigarettes weekly than with 10–49 cigarettes weekly, but this did not reach statistical significance (Table III).

The opportunity was taken using all the available data collected by Stocks (1958, 1970) on non-gastrointestinal cancers to examine smoking habits in relation to coffee and tea consumption. There was no significant relationship between smoking habits and coffee consumption, though among women (but not men) heavy tea drinkers were more often smokers. Among 551 women who drank 5 or more cups of tea daily 23% were smokers,

**Table III** Relative risk of pancreas cancer by smoking habits

| *Smoking habits* | | *Cases* | *Controls* | *Relative risk (95% confidence limits)* |
|---|---|---|---|---|
| *Males* | | | | |
| | Non smoker | 16 | 42 | 1.00 |
| 10–49 | Cigarettes per week | 13 | 26 | 1.30 (0.52–3.21) |
| 50–149 | Cigarettes per week | 47 | 78 | 1.59 (0.80–3.14) |
| +150 | Cigarettes per week | 7 | 18 | 1.05 (0.36–3.03) |
| | Pipe only | 26 | 54 | 1.23 (0.59–2.59) |
| *Females* | | | | |
| | Non smoker | 87 | 184 | 1.00 |
| 10– 49 | Cigarettes per week | 8 | 16 | 1.09 (0.41–2.91) |
| 50–149 | Cigarettes per week | 10 | 14 | 1.60 (0.63–4.07) |
| *Both sexes* | | | | |
| | Non smoker | 103 | 226 | 1.00 |
| 10– 49 | Cigarettes per week | 21 | 42 | 1.26 (0.66–2.44) |
| 50–149 | Cigarettes per week | 57 | 92 | 1.66 (0.97–2.85) |
| +150 | Cigarettes per week | 8 | 18 | 1.24 (0.48–3.23) |
| | Pipe only | 27 | 54 | 1.32 (0.68–2.57) |

compared to 17% of 791 who drank <5 cups daily.

In contrast to the findings of MacMahon *et al.* (1981), this study finds no evidence of a positive relationship between pancreas cancer and coffee consumption. The difference cannot be explained in terms of the choice of controls since a positive result was obtained in the U.S. study when controls were chosen with cancer as well as with other diseases. The exposed group was larger in the U.S. study, though the reference group of non-coffee drinkers was larger in the present study (124 cases and 244 controls compared to 20 and 88 respectively in the U.S. study). It might be argued that we were unlikely to detect the relationship because coffee consumption was so low in our study group (only 1 in 5 controls drank coffee daily). This view, however, would ignore the fact that, compared to non-coffee drinkers, a significant excess of pancreas cancer was found in the U.S. study among those who drank only 1 or 2 cups of coffee daily. But in fact no suggestion of such an excess was found in our study.

Certain findings in the present study confirm previous observations. More heavy tea drinkers smoked cigarettes than did modest tea drinkers (Stocks, 1958). A positive relationship was found between smoking and pancreas cancer, though this was not statistically significant, similar to that reported in 3 other case-control studies (Wynder *et al.*, 1973; Lin & Kessler, 1981; and MacMahon *et al.*, 1981).

The most noteworthy finding in the present study is the positive relationship observed between pancreas cancer and tea consumption. This cannot be explained in terms of social class since although tea consumption shows a marked relationship with social class, pancreas cancer in the period in question does not (Registrar General, 1958). This finding is unexpected, and indeed in the U.S. study, a slight inverse relationship with tea was noted. If, as seems likely, tea and coffee consumption are inversely correlated, a positive relationship with tea may conceivably have failed to emerge in the U.S. study because the coffee effects on pancreas cancer risk dominated the findings with respect to tea, it being relevant that tea drinkers were outnumbered in that study by coffee drinkers.

Coffee and tea have, of course, constituents in common such as caffeine, but if this was the relevant agent the absence of a positive relationship with coffee in the present study is difficult to explain. Although it is conceivable that the discrepancy in findings concerning coffee is due to differences between the coffee drunk in Britain in the 1950s (much of it instant coffee or coffee extract with chicory) and the mainly ground coffee drunk in the U.S., it is difficult to think of relevant constituents common to ground coffee and tea but absent from soluble coffee. It would certainly be surprising if, in a study prompted by the hypothesis about coffee, we had stumbled on a causual relationship between tea and this cancer, particularly since tea consumption and pancreas cancer mortality in different countries are not positively correlated (Stocks, 1970). The present findings weigh against the hypothesis of a causal relationship between coffee and pancreas cancer, as also do the findings of 5 of 6 other studies (Goldstein, 1982; Jick & Dinan, 1981; Severson *et al.*, 1982; Nomura *et al.*, 1981; Kessler, 1981; and Heuch *et al.*, 1983).

It is possible that the present findings and also those of the first U.S. study reflect different effects on beverage consumption of pancreas cancer compared to those of other disorders. However, it may not be a coincidence that the positive findings in each of the studies under discussion concerns the popular national (non-alcoholic) beverage – coffee in the U.S.A. and tea in the Britain of the 1950s. The discrepancy between the two studies would be explained if the positive findings in each reflected a relationship between pancreas cancer and some factor connected with the popular national beverage rather than with the beverage itself. By this we do not necessarily mean something as clearly linked to a beverage as an additive such as sugar or saccharine (in fact both seem very unlikely), but more indirect factors since, conceivably, the lifestyle of those who habitually avoid these beverages may incidentally involve less exposure to the relevant agent than the average.

**Addendum** (Added in proof)

Since the above was submitted data has emerged from a prospective study suggesting that pancreas cancer may produce a non-specific and slight increase in fluid consumption, presumably by impairing glucose tolerance and thereby causing some compensatory thirst. This may account for the findings concerning tea in the present study, (Kinlen *et al.*, *Lancet* (in press)).

We are most grateful for the assistance of Tony Willows, Lesley Jones and Helena Blain. L.J. Kinlen is also grateful for the help given by the many people approached on behalf of the Cancer Research Campaign for information about the records of the study carried out by the late Dr Percy Stocks while holding a British Empire Cancer Campaign Fellowship, and particularly to Dr Davies and the staff of the Biochemistry Department of Bangor University who found some of these records. L.J.K. Gibb is a fellow of the Cancer Research Campaign.

**References**

BRESLOW, N.E., DAY, N.E., HALVORSEN, K.T., PRENTICE, R.L. & SABAI, C. (1978). Estimation of multiple relative risk functions in matched case-control studies. *Am. J. Epidemiol.*, **108**, 299.

GOLDSTEIN, H.R. (1982). No association found between coffee and cancer of the pancreas. *N. Engl. J. Med.*, **306**, 997.

HEUCH, I., KVALE, G., JACOBSEN, S.K. & BJELKE, E. (1983). Use of alcohol, tobacco and coffee and risk of pancreatic cancer. *Br. J. Cancer*, **48**, 637.

JICK, H. & DINAN, B.J. (1981). Coffee and pancreatic cancer. *Lancet*, **ii**, 92.

KESSLER, I.I. (1981). Coffee and cancer of the pancreas. *N. Engl. J. Med.*, **304**, 1605.

LIN, R.S. & KESSLER, I.I. (1981). A multifactorial model for pancreatic cancer in man: epidemiologic evidence. *JAMA*, **245**, 147.

MACMAHON, B., YEN, S., TRICHOPOULOS, D., WARREN, K. & NARDI, G. (1981). Coffee and cancer of the pancreas. *N. Engl. J. Med.*, **304**, 630 and 1606.

NOMURA, A., STEMMERMANN, G.N. & HEILBRUN, L.K. (1981). Coffee and pancreatic cancer. *Lancet*, **ii**, 415.

REGISTRAR GENERAL. (1958). The Registrar General's Decennial Supplement, England and Wales, 1951, *Occupational Mortality Tables*, London, HMSO.

SEVERSON, R.K., DAVIS, S. & PALISSAR, L. (1982). Smoking, coffee and cancer of the pancreas. *Br. Med. J.*, **285**, 214.

STOCKS, P. (1958). Cancer incidence in North Wales and Liverpool region in relation to habits and environment. Suppl. to Pt II. *Br. Empire Cancer Campaign 35th Ann. Rep.* (1957).

STOCKS, P. (1970). Cancer mortality in relation to national consumption of cigarettes, solid fuel, tea and coffee. *Br. J. Cancer*, **24**, 215.

TRICHOPOULOS, D., PAPAPOSTOLOU, M. & POLYCHRONOPOULOU, A. (1981). Coffee and ovarian cancer. *Int. J. Cancer*, **28**, 691.

WYNDER, E.L., MABUCHI, K., MARUCHI, N. & FORTNER, J.G. (1973). Epidemiology of cancer of the pancreas. *J. Natl Cancer Inst.*, **50**, 645.